# Black Bean Anthocyanin-Rich Extract from Supercritical and Pressurized Extraction Increased *In Vitro* Antidiabetic Potential, While Having Similar Storage Stability

**DOI:** 10.3390/foods9050655

**Published:** 2020-05-19

**Authors:** Ming Hsieh-Lo, Gustavo Castillo-Herrera, Luis Mojica

**Affiliations:** Tecnología Alimentaria, Centro de Investigación y Asistencia en Tecnología y Diseño del Estado de Jalisco, A.C. CIATEJ, Unidad Zapopan, El Bajío, 45019 Zapopan, Jalisco, Mexico; minghsiehlo@gmail.com (M.H.-L.); gcastillo@ciatej.mx (G.C.-H.)

**Keywords:** anthocyanins-rich extract, black bean, supercritical fluid extraction, pressurized liquid extraction, antidiabetic, antioxidant, storage stability

## Abstract

Black bean is a source of anthocyanins and other phenolic compounds that are associated with health benefits. This work aimed to optimize the extraction and determine the stability and biological potential of black bean anthocyanin-rich extracts recovered by supercritical fluid extraction (SFE) and pressurized liquid extraction (PLE). The highest concentration of anthocyanins and total phenolic compounds were recovered with SFE using 300 bar, 60 °C and co-solvent ethanol/distilled water (50/50, *v*/*v*). Eleven non-colored phenolic compounds were identified in SFE extract using Ultra performance liquid chromatography - Electrospray ionization–Quadrupole -Time of flight - Mass spectrometry (UPLC-ESI-QToF-MS/MS). Myricetin, syringic acid, rutin hydrate and chlorogenic acid presented the highest relative area among identified compounds. Compared to leaching extraction, SFE extracts showed a similar storage stability at 4, 25 and 32 °C (*p* < 0.05), but with a higher antioxidant potential (2,2-diphenyl-1-picryl-hydrazil (DPPH) IC_50_: 0.078 ± 0.01; 2,2’-azino-bis(3-ethylbenzothiazoline)-6-sulfonic acid (ABTS) IC_50_: 0.161 ± 0.03) and antidiabetic potential (α-amylase IC_50_: 124.76 ± 12.97; α-glucosidase IC_50_: 31.30 ± 0.84; dipeptidyl peptidase-IV IC_50_: 0.195 ± 0.01). SFE extraction is an efficient method to obtain anthocyanins and other phenolic compounds with exceptional biological potential.

## 1. Introduction

Common bean (*Phaseolus vulgaris* L.) is one of the most commonly consumed legumes worldwide. In 2016, Mexico produced 1.08 million tons of common bean, which is 5.5% of world production. It is an inexpensive source of proteins, minerals, vitamins and bioactive compounds such as phenolic compounds, including anthocyanins [1]. Anthocyanins are found mainly in the seed coat of dark-colored common bean. They are water-soluble flavonoids constituted by an anthocyanidin (flavylium ring), sugars, and optionally acylation groups [2]. Anthocyanins have potential to be used as food colorants, due to their attractive blue/red colors and non-toxicity. Moreover, these molecules are associated with potential health benefits, being able to provide anti-inflammatory, antioxidant and antidiabetic effects [2].

Type 2 diabetes mellitus (T2DM) is a noncommunicable metabolic disorder, characterized by the presence of chronic hyperglycemia, due to inadequate production of insulin and defect in insulin sensitivity [3,4]. Hyperglycemia in T2DM leads to the overproduction of free radicals, resulting in increased oxidative stress. The accumulation of free radicals and reactive oxygen species (ROS) can cause damage to macromolecules such as DNA, protein, and membranes and lead to severe complications such as kidney failure, liver dysfunction, blindness, heart attack and nerve damage [3].

Studies suggest that the beneficial effects of anthocyanins are attributed to their capacity to inhibit the formation of ROS and the activity of carbohydrate hydrolyzing enzymes, such as α-amylase and α-glucosidase, contributing to the prevention and treatment of T2DM [3,5]. Furthermore, glycemic control can be improved by the potential of anthocyanins to inhibit dipeptidyl peptidase-IV (DPP-IV), thereby avoiding the degradation of incretin hormones [6]. GIP (glucose-dependent insulinotropic polypeptide) and GLP-1 (glucagon-like peptide 1) are incretin hormones with therapeutic potential on patients with T2DM, since they can increase the insulin release and decrease the glycogen release [7].

However, anthocyanins are very unstable and susceptible to degradation. In this regard, their stability can be enhanced through copigmentation, which is a supramolecular complexation. This non-covalent association occurs between anthocyanin and a copigment by intramolecular or intermolecular interactions. The most commonly used copigments are flavonoids, tannins, phenolic acids, amino acids, and other anthocyanins [5,8]. Therefore, using efficient extraction methods could significantly increase anthocyanins and other phenolic compounds’ concentration, thereby improving the copigmentation effect.

Pressurized liquid extraction (PLE) and supercritical fluid extraction (SFE) are alternative methods to conventional solid-liquid extractions, which can greatly reduce the amount of employed solvents and sample processing times. PLE utilizes liquid solvent at high temperature and high pressure, providing higher extraction efficiency, due to greater solubility and solvent penetration in the solid matrix [9,10]. On the other hand, SFE uses carbon dioxide above its critical point. The carbon dioxide could increase anthocyanins and other phenolic compounds yield from common beans, due to its ability to dissolve as a liquid and to penetrate the matrix like a gas [11].

Although the recovery of anthocyanin-rich extract from blueberry, elderberry, cranberry and other sources through PLE and SFE has been investigated, there are no reports of PLE and SFE of whole black bean anthocyanins. In this context, this work aimed to (i) optimize the extraction of anthocyanins and polyphenols from black beans using PLE and SFE techniques, and (ii) evaluate the stability and antidiabetic potential of anthocyanin-rich extracts. Our hypothesis was that SFE and PLE techniques would recover more anthocyanins and other phenolic compounds than conventional leaching extraction, leading to greater stability and biological potential.

## 2. Materials and Methods

### 2.1. Materials

Black bean (*Phaseolus vulgaris* L.) “San Luis” cultivar was obtained from local sellers in the city of Sombrerete, Zacatecas, Mexico in 2017. Ethanol (95%), (+)-catechin hydrate (98%), chlorogenic acid (95%), myricetin (99%), ferulic acid (98%), kaempferol (90%), quercetin (95%), syringic acid (95%), epicatechin (90%), p-coumaric acid (98%), rutin hydrate (94%), acetonitrile (99%), formic acid (99%), methanol (99%), chlorhydric acid (95%), citric acid monohydrate (>98%), potassium chloride (95%), ethylenediaminetetraacetic acid (99%), sodium acetate (95%), sodium carbonate (>98%), gallic acid (97.5%), 1,1-diphenyl-2-picryl-hydrazil (DPPH) (>98%), 2,2’-azino-bis(3-ethylbenzothiazoline)-6-sulfonic acid (ABTS) (98%), 6-hydroxy-2, 5, 7, 8-tetramethylchrome-2-carboxylic acid (Trolox^®^) (97%), α-Amylase from porcine pancreas (EC3.2.1.1), α-glucosidase from *Saccharomyces cerevisiae* (EC3.2.1.20), acarbose (≥95%), dipeptidyl peptidase IV human enzyme, 3,5-dinitrosalicylic acid (≥98%), p-4-nitrophenyl-α-D-glucopyranoside (≥99%), soluble starch, phosphate buffered saline, folin-ciocalteu reagent, sitagliptin phosphate, acetic acid (99%), delphinidin 3-*O*-glucoside (≥98%), malvidin 3-*O*-glucoside (≥98%) and cyanidin 3-*O*-glucoside (≥98%) were purchased from Sigma-Aldrich (St. Louis, MO, USA) and Fisher Scientific (Pittsburgh, PA, USA). DPP-IV Glo^®^ protease assay was purchased from Promega (Madison, WI, USA).

### 2.2. Pressurized-Liquid Extraction Procedure (PLE)

PLE was performed using a modified Thar^®^ SFE500 extractor (Thar Process, Pittsburgh, PA, USA), equipped with stainless steel extraction cells (500 mL volume). In addition, 75.8 ± 1.0 g of whole black beans were weighed and placed in the extraction cell. Prior to each extraction, the extraction cell was heated until the process temperature is reached. Based on the experimental design, the extraction cell was filled with the appropriate extraction solvent, and pressurized and heated under controlled PLE conditions. A period of 15 min was employed, to carry out all the extractions of the experimental design.

Temperatures of 40, 50 and 60 °C, and pressures of 100, 200 and 250 bars were evaluated. Also, acidified distilled water, ethanol-distilled water 50% (v/v) and ethanol-distilled water 70% (v/v) were used as extraction solvents, and citric acid monohydrate was used to adjust the pH to 2.0 ± 0.1. Obtained extracts were transferred to an amber container and stored at −20 °C until analysis.

### 2.3. Supercritical CO_2_ Extraction Procedure (SFE)

SFE was performed using a Thar^®^ SFE500 extractor (Thar Process, Pittsburgh, PA, USA), equipped with stainless steel extraction cells (500 mL volume). For the experiment, 50 ± 1.0 g of whole black beans were weighed and placed in the extraction cell mixed with 20 ± 1.0 g of glass beads. Then, the gas carbon dioxide was pressurized and introduced at a flow of 10 g/min with 10% of the appropriate co-solvent. It was pressurized to the desired pressure and heated to the specified temperature, in order to reach the supercritical state; all extractions of the experimental design were carried out for 60 min.

For SFE, temperatures of 40, 50 and 60°C, and pressures of 160, 200 and 300 bars were evaluated. Also, acidified distilled water, ethanol-distilled water 10% (v/v) and ethanol-distilled water 50% (v/v) were used as extraction co-solvents, and citric acid monohydrate was used to adjust the pH to 2.0 ± 0.1. Obtained extracts were transferred to an amber container and stored at −20 °C until analysis.

### 2.4. Leaching Process (LEA)

The extractions were performed as reported by [1], with some modifications. In essence, 0.1 g of common bean coat or 1.18 g of triturated black bean (equivalent to 0.1 g of bean coat) were extracted with 10 mL of acidified ethanol (ethanol in 1 N HCl, 85:15, v/v). The solution was mixed and shaken for 30 min, centrifuged at 10,000 RPM for 15 min, and the supernatant was made up to 10 mL volume with acidified ethanol. The extracts were stored at −20 °C until analysis.

### 2.5. Identification of Anthocyanins by UHPLC

The samples were analyzed by ultra-high-performance liquid chromatography (UHPLC), using a Waters Acquity Ultra-performance liquid chromatography (UPLC) H-Class system (Milford, MA, USA), equipped with a vacuum degasser, a quaternary pump, an autosampler and a column heater. Chromatographic separation was carried out at 45 °C on a Waters Cortecs UPLC C18 column (4.6 × 50 mm, 2.7 μm). A mobile phase with two eluent solvents were used: eluent (A) acetonitrile and eluent (B) 5% methanol and 0.1% formic acid in Mili-Q water. The gradient elution was as follows: 0–2% A at 0–2 min, 2–7% A at 2–5 min, 7–13% A at 5–7 min, 13–20% A at 7–9 min, 20–55% A at 9–11.5 min, 55–90% A at 11.5–13.5 min, 90% A at 13.5–14.5 min, 90–3% A at 14.5–14.95 min, 3% A at 14.95–18 min, and 3–0% A at 18–20 min. The flow rate was kept at 0.25 mL/min, and the injection volume was 10 μL. The eluate was monitored at 520 nm on the PDA.

### 2.6. Identification of Non-Colored Phenolic Compounds by UPLC-ESI-QToF-MS/MS

Chromatographic analysis was performed on a Waters Acquity UPLC H-Class system (Milford, MA, USA), equipped with a vacuum degasser, a quaternary pump, an autosampler and a column heater. Chromatographic separation was carried out at 40 °C with a flow rate of 0.3 mL/min on an Acquity UPLC BEH C18 column (2.1 × 50 mm, 1.7 μm). A mobile phase with two eluent solvents were used: eluent (A) 0.3% formic acid in acetonitrile and eluent (B) 0.3% formic acid in Mili-Q water. The gradient elution was as follows: 90% A at 0–1 min, 90–30% A at 1–11 min, 30–90% A at 11–12 min, 90% at 12–15 min. The injection volume was set at 8 μL.

A mass spectrometry (MS) analysis was performed on a Water Xevo G2-XS QToF quadrupole time-of-flight mass spectrometry, equipped with an electrospray ionization (ESI) interface (Milford, MA, USA). The MS acquisition was operated in negative ion mode, and mass range was set at m/z 50 to 800. The parameters were set as follows: capillary voltage: 2.5 kV, cone voltage: 40 kV, source temperature: 100 °C, desolvation temperature: 250 °C, collision energy: 6.0 eV. MassLynx 4.1 MS software (Milford, MA, USA) was used to control the instruments and to process the data.

### 2.7. Determination of Total Phenolic Content (TPC) and Total Anthocyanins

TPC was quantified using the Folin–Ciocalteau method described by [1]. First, 50 μL of bean coat extract, blank (distilled H_2_O) or gallic acid standard (calibration curve, 40–200 μg/mL) was placed in a 96-well microplate. Afterward, 50 μL of Folin–Ciocalteu 1N reagent was added, mixed, and allowed to stand for 5 min before the addition 100 μL of 20% Na_2_CO_3_. Finally, it was allowed to stand for 10 min before reading at an absorbance of 690 nm with a UV-visible spectrophotometer. The results were expressed as mg equivalents of gallic acid per gram of bean coat (mg GAE/g coat).

Total anthocyanins were determined by pH differential method (AOAC Official Method 2005.02). Aliquots of extracts were diluted to a factor of 1:10 v/v with potassium chloride (pH 1.0, 0.025 M) and sodium acetate (pH 4.5, 0.4 M). Samples diluted with each pH were transferred to a 96-well microplate and absorbance was read at 520 and 700 nm, using a UV-visible spectrophotometer. The total anthocyanins were expressed as mg of cyanidin-3-glucoside equivalents per gram of bean coat (mg C3GE/g coat).

### 2.8. Antidiabetic Potential

#### 2.8.1. α-Amylase and α-Glucosidase Inhibition

The inhibition of the enzymes was measured using the method described by [1]. For α-amylase, 50 μL of bean coat extract, negative control (distilled H_2_O) or positive control (1 mM acarbose) was placed in 2 mL Eppendorf^®^ tubes. For the blank, 50 μL of 0.02 M sodium phosphate buffer (PBS) pH 6.9 were added, and for the samples, 50 μL of 13U/mL α-amylase solution (type VI-B from porcine pancreas in 0.02 M PBS pH 6.9). After that, the tubes were mixed and incubated for 10 min at 37 °C, before 50 μL of 1% soluble starch (previously dissolved in PBS and boiled for 10 min) was added to each tube and incubated for another 10 min. Finally, 100 μL of dinitro salicylic acid reagent was added, and the tubes boiled for 5 min. The mixture was diluted with 1 mL of distilled water and absorbance read at 540 nm. Results were presented as IC_50_ values (mg C3GE/g of bean coat).

For α-glucosidase, 50 μL of bean coat extract, negative control (distilled H_2_O) or positive control (1 mM acarbose) was placed in a 96-well plate. For the blank, 50 μL of 0.1 M sodium phosphate buffer (PBS) pH 6.9 were added, and for the samples, 50 μL of 1U/mL α-glucosidase solution (in 0.1 M PBS pH 6.9). After that, the microplate was incubated for 10 min at 37 °C, before 50 μL of 5 mM pnitrophenyl-α-D-glucopyranoside solution (in 0.1 M PBS pH 6.9) was added to each well and incubated for another 5 min. Finally, the absorbance was read at 405 nm. Results were presented as IC_50_ (mg C3GE/g of bean coat).

#### 2.8.2. Dipeptidyl PeptIdase IV (DPP-IV) Inhibition

DPP-IV inhibition was measured by the DPP-IV Glo^®^ Protease Assay. A 40 μL negative control (distilled H_2_O), 40 μL positive control (5 μM sitagliptin) or 40 μL bean coat extract, was mixed with 50 μL of DPP-IV reagent and placed in a white-walled 96-well plate. DPP-IV human enzyme was prepared in buffer (100 mM Tris, 200mM NaCl, 1 mM EDTA, pH 8.0), at a concentration of 10 ng/mL. The blank contained only buffer and DPP-IV reagent, while the control and the samples contained DPP-IV reagent and 10 μL purified DPP-IV human enzyme. Luminescence was measured after mixing and incubating for 30 min at 27 °C, using a SpectraMax^®^ i3 Multi-Mode Detection Platform (Molecular Devices, Sunnyvale, CA, USA). Percentage of inhibition was calculated from the blank and enzyme control for each sample. Results were presented as IC_50_ (mg C3GE/g of bean coat).

### 2.9. Antioxidant Capacity

The antioxidant capacity of the samples was measured by 2,2-diphenyl-1-picryl-hydrazil stable radical (DPPH∙) and 2,2’-azino-bis(3-ethylbenzothiazoline)-6-sulfonic acid (ABTS).

#### 2.9.1. DPPH

For DPPH, 20 μL of bean coat extract, blank (distilled H_2_O) or trolox^®^ standard (calibration curve, 0.01–0.25 mM) was placed in a 96-well microplate. Then, 180 μL of DPPH (2.36 mg DPPH/100 mL of ethanol-water 80:20, v/v) was added, mixed and allowed to stand for 30 minutes, before reading at an absorbance of 517 nm with a UV-visible spectrophotometer. The results were expressed as IC_50_ values (mg C3GE/g coat).

#### 2.9.2. ABTS

For ABTS, the free radical was generated by mixing 100 mL H_2_O with 3.5 mM ABTS and 1.225 mM Na_2_S_2_O_8_ and allowing to stand for 12–16 h at room temperature and in darkness. Prior to the analysis, the solution was adjusted with water to an absorbance of 0.7 ± 0.02 at 734 nm. Then, 20 μL of bean coat extract, blank (distilled H_2_O) or trolox^®^ standard (calibration curve, 0.01–0.25 mM) was placed in a 96-well microplate. Afterward, 180 μL of ABTS was added, mixed and read at an absorbance of 734 nm with a UV-visible spectrophotometer. The results were expressed as IC_50_ values (mg C3GE/g coat).

### 2.10. Anthocyanins and Color Stability Evaluation

Anthocyanins concentration, obtained by LEA and SFE, and color stability, were analyzed in flavored transparent water (Bonafont^®^ Levité Fresa-Lychee) through two different experiments, in which the effects of light and storage were evaluated. In the experiment of the effect of light exposure, samples in transparent tubes were exposed to white fluorescent light for 10 days at 32 °C in an incubator, sampling every two days. The controls were samples in transparent tubes covered with aluminum foil and stored at 32 °C. The experiment of the effect of storage was performed at 4 °C, 25 °C and 32 °C for 6 weeks in dark conditions, sampling every week. The concentration of the initial anthocyanin solutions was adjusted to 3.5 ± 0.1 mg C3GE/L and the initial pH to 1.86. The pH was monitored throughout both experiments.

### 2.11. Color Measurements

The color was measured using a CM-5 Spectrophotometer (Konika Minolta Sensing Americas, Inc. Ramsey, NJ, USA). The instrument was calibrated with distilled water and the following parameters were used: L*, a* and b*; observer/illuminant: 10° and D65 and path length: 1cm. After this, 10 ± 1.0 mL of sample were placed in a 10 mm plastic cell and the color parameters (L*, a* and b*) were measured and recorded. Furthermore, ΔE* was calculated using the following Equation (1):

(1) $$\Delta E^{*}=\left[\left(\Delta L^{*}\right)2+\left(\Delta a^{*}\right)2+\left(\Delta b^{*}\right)2\right]1/2$$

### 2.12. Reaction Kinetics of Anthocyanins

The anthocyanin degradation in aqueous solutions follows first-order kinetics [2,5]. The first-order reaction rate constants (*k*), half-lives (*t*_1/2_), energy of activation (*E_a_*) and the change in the reaction rate constant for 10°C (*Q*_10_), were calculated using the following Equations (2) and (3):

(2) $$\ln A_{t}=A_{0}-kt;\;t_{1/2}=-\ln0.5\cdot k^{-1}$$

(3) $$\ln k=\ln A-\frac{E_{a}}{R}\left(\frac{1}{T}\right);\;Q_{10}=e^{\left(\frac{E_{a}}{R}\right)\left(\frac{10}{T_{2}T_{1}}\right)}$$

where *A_t_* is the total monomeric anthocyanin at time *t*, *A*_0_ is the total monomeric anthocyanin at time zero; *k* is the reaction constant in days^−1^; *t* is the time in days; *A* is the Arrhenius pre-exponential factor; *E_a_* is the activation energy (kJ/mol); *R* is the gas constant (8.314 J/mol-K), and *T* is the temperature in Kelvin.

### 2.13. Statistical Analysis

Response surface methodology was used to optimize the extraction of anthocyanin and polyphenols. Experiments were established based on a D-optimal design experiment with the quadratic model and a total of 22 runs. Optimization experimental data was analyzed using Stat-Ease Design Expert 10.0.1.0 (Stat-Ease Inc., Minneapolis, MN, USA). A one-way ANOVA was used to evaluate the observed data and the regression coefficients of linear, quadratic, and interaction terms, and their effects were generated and analyzed. All the terms in the model were tested statistically using F-test (*p* < 0.05).

The assays were run in triplicate and performed in independent replicates. The data obtained was analyzed using one-way ANOVA by StatPoint STATGRAPHICS Centurion XVI 16.1.03 statistical software (StatPoint Technologies, Inc., Warrenton, VA, USA). Statistical differences among independent variables were determined using Tukey’s Posthoc Test (*p* < 0.05). The differences between the mean of two samples were determined using a two-sample *t*-test (*p* < 0.05). The correlation graphs were created using GraphPad Prism software 7.00 (GraphPad Software, Inc., San Diego, CA, USA).

## 3. Results and Discussion

### 3.1. Effect of Pressurized Liquid Extraction and Supercritical Fluid Extraction on Anthocyanins

**Figure 1:**
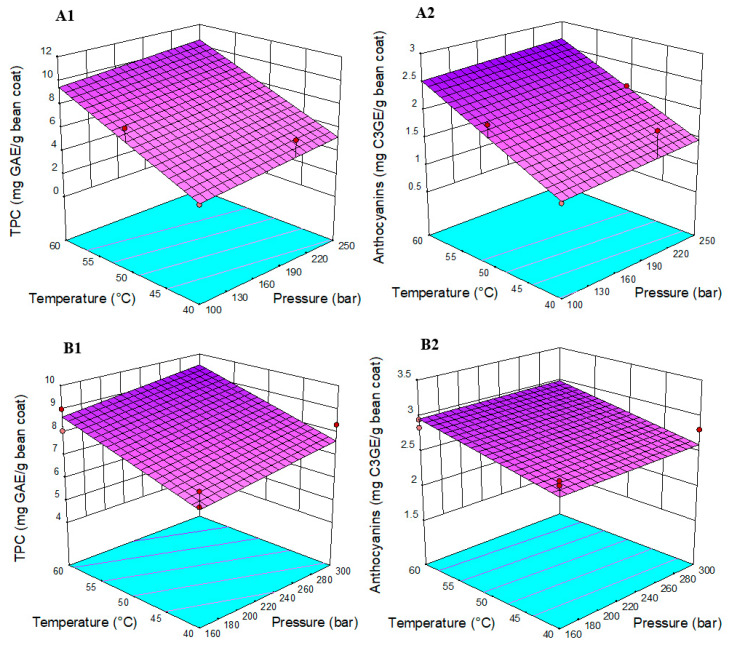
Response surface model for (**A**) Pressurized Liquid Extraction and (**B**) Supercritical Fluid Extraction, showing the effects of temperature and pressure on (1) anthocyanins and (2) total phenolic compounds. C3GE: Cyanidin 3-glucoside equivalents, GAE: Gallic acid equivalents.

The whole experimental design matrix employed to optimize PLE with the independent factors and their anthocyanin and TPC yield can be seen in Table S1. The analysis of the response surface model showed that the recovery of anthocyanin and TPC was affected mainly by the temperature and the co-solvent used (Figure 1A1,A2). The highest predicted concentration of anthocyanin and total phenolic compounds with PLE was 2.67 mg C3GE/g bean coat and 10.59 mg GAE/g bean coat, respectively, using 250 bar, 60 °C and, as co-solvent, ethanol-water 50%.

For SFE, Table S2 shows the employed experimental conditions and their anthocyanin and TPC yield. As in the case of PLE, only the combination of temperature and co-solvents effects had a significant impact on the extraction yield (Figure 1B1,B2). The optimal conditions for the extraction of anthocyanins with SFE were 300 bar, 60 °C and, as co-solvent, ethanol-water 50% v/v. The predicted concentration of anthocyanins with the D-optimal design was 2.99 mg C3GE/g bean coat and TPC of 9.49 mg GAE/g bean coat.

The increase in temperature favors the extraction of phenolic compounds. This is because high temperatures allow better penetration of the solvent to the matrix and greater solubility of the phenolic compounds in the solvent, by reducing the solvent’s viscosity and surface tension [9,12,13,14]. In this work, moderate temperatures were considered to avoid the degradation of anthocyanins, which are thermolabile. Further increases in temperature (>60–70 °C) decrease the recovery of bioactive compounds, due to the decomposition of volatile components and the reduction in the density of CO_2_ [9,13].

For the co-solvent mixtures, the combination of water and ethanol as a co-solvent was suitable for the extraction of anthocyanin, but may be different for the recovery of other phenolic compounds. An increase in ethanol concentration improves the recoveries of anthocyanins, due to an enhanced capacity of ethanol to form hydrogen bonds and dipole-dipole interactions with phenols. As a result, the dissolving capacity of the phenolic compounds in the co-solvent is improved [10,12]. However, the addition of water on the recovery of anthocyanins and other phenolic compounds has special importance. The solubility of anthocyanins is increased in aqueous solutions. Moreover, the formation of carbonic acid by the solubilization of CO_2_ decreases the pH of the solvent. At low pH, anthocyanins are predominantly present in their flavylium cation form, making them more hydrophilic, thus facilitating their extraction [13]. Therefore, ethanol can enhance anthocyanins solubility, while the water contributes to the desorption of the solute from the matrix [15]. This explains why the highest recovery of anthocyanin was obtained with EtOH–H_2_O 50% v/v, rather than EtOH–H_2_O 70% v/v, EtOH–H_2_O 10% v/v, or acidified water. Combinations of CO_2_, ethanol and water have also been reported to be efficient to extract anthocyanins and phenolics from blueberry, elderberry and cranberry [10,12].

In this work, there was no significant effect of pressure within the ranges studied for anthocyanin extraction. These findings are in agreement with [12]. This can be an advantage to keep the pressure as low as possible for economic saving. Nevertheless, the extraction yield is improved by the presence of pressure, which can strengthen the interaction between the fluid and matrix, by increasing the fluid density and decreasing the distance between the molecules [13].

The acidification of the co-solvent also increases the efficiency of the recovery of anthocyanins. Low pH solvents can significantly increase the yield of anthocyanins and other phenolics, due to the disruption of cell membranes and the molecular bonds of sugar, acyl, aryl and alkyl [10,14,15,16]. However, high concentrations of citric acid in anthocyanin-rich extracts could negatively affect the integrity of the compounds of interest [17].

### 3.2. Comparison between Conventional and Alternative Extraction Methods

Based on the results of the supercritical fluid and pressurized liquid experiments, extractions of anthocyanins were conducted at the respective optimum point, in order to compare it with the conventional leaching extraction of manually husked bean coat (LEA-M) and whole black bean (LEA-WB). As shown in Table 1, the concentration of anthocyanin obtained by SFE, PLE and LEA-M were the highest and statistically equal (*p* < 0.05), while the lowest concentration was obtained by LEA-WB. Regarding the total phenolic compounds, the highest concentration recovered was SFE, with 11.09 mg GAE/g bean coat, followed by LEA-M with 8.92. Similar to the case of anthocyanins, the lowest concentration of TPC was found in LEA-WB. Since only the concentration of TPC recovered by SFE was higher than the leaching extraction of bean coat, our hypothesis was partially supported by these results.

The loss of anthocyanin and other phenolic compounds in LEA-WB may be due to the interaction of the flavonoids with cotyledon proteins. This interaction not only affects the anthocyanin availability, but also has a negative influence on the bioactivity and antioxidant potential of anthocyanins, due to the loss of hydroxyl groups upon the formation of covalent/non-covalent interactions between flavonoids and proteins [18]. Although anthocyanin concentrations were statistically the same for LEA-M, SFE and PLE, alternative methods can offer important advantages (Table S3). Unlike LEA-M, SFE and PLE do not require sample processing such as husking and grinding, allowing faster extractions by saving a considerable amount of time. In addition, the solvent used for the extraction of anthocyanin with these techniques is GRAS (generally recognized as safe) solvents [15]. Using alternative methods, it is possible to extract insoluble-bound phenolics, which can improve the anthocyanins stability through co-pigmentation [5,19]. Additionally, the remnant of the extracted black bean could be used as a food ingredient, due to its protein and complex carbohydrate content. Moreover, SFE can be an extraction method that is even more promising than PLE, since it reduces solvent consumption significantly, as shown in Table S3. Furthermore, it has been reported that SFE is a more selective method than PLE for the extraction of anthocyanins [14].

### 3.3. Anthocyanins in Common Beans Cultivar San Luis

Three anthocyanins were identified by UHPLC: petunidin 3-*O*-glucoside, delphinidin 3-*O*-glucoside and malvidin 3-*O*-glucoside, which were found in all samples. The proportion of the three anthocyanins in each analyzed extract was statistically similar (SFE, PLE, LEA-M and LEA-WB). Delphinidin 3-*O*-glucoside is the anthocyanin with the highest proportion, followed by petunidin 3-*O*-glucoside and malvidin 3-*O*-glucoside, with a relative area of 58.37%, 30.92% and 10.69%, respectively (Figure S1). These anthocyanins have been identified in other black common bean cultivars [1]. The extraction yield of anthocyanins is affected by the employed solvent, but the proportion of the three anthocyanins remains the same, suggesting that the anthocyanins profile might depend more on the cultivar, rather than the extraction method.

### 3.4. Non-Colored Phenolic Compounds in Common Beans Cultivar San Luis

Eleven non-colored phenolic compounds were identified in the anthocyanin-rich extracts by Ultra performance liquid chromatography—Electrospray ionization—Quadrupole—Time of flight—Mass spectrometry (UPLC-ESI-QToF-MS/MS) (Table 2).

The identification of the compounds was based on the retention time of pure standards and comparing their m/z negative ionization values. Catechin, syringic acid and myricetin were the major phenolic compounds found in the extract by LEA-M, while in LEA-WB, rutin hydrate was detected, in addition to the other aforementioned compounds. Chlorogenic acid, syringic acid, rutin hydrate, myricetin, quercetin, kaempferol and ferulic acid were the major compounds detected in the anthocyanin extract recovered by SFE and PLE. The different chemical profiles might be due to the different extraction methods used.

### 3.5. Biological Potential of the Anthocyanin-Rich Extracts

In order to assess the bioactive properties of the anthocyanin-rich extracts obtained by SFE and PLE, this work evaluated the inhibition capacity of the enzymes α-amylase, α-glucosidase and DPP-IV by biochemical assays and the free radical scavenging activities by ABTS and DPPH radical assays.

#### 3.5.1. Antidiabetic Potential

**Figure 2:**
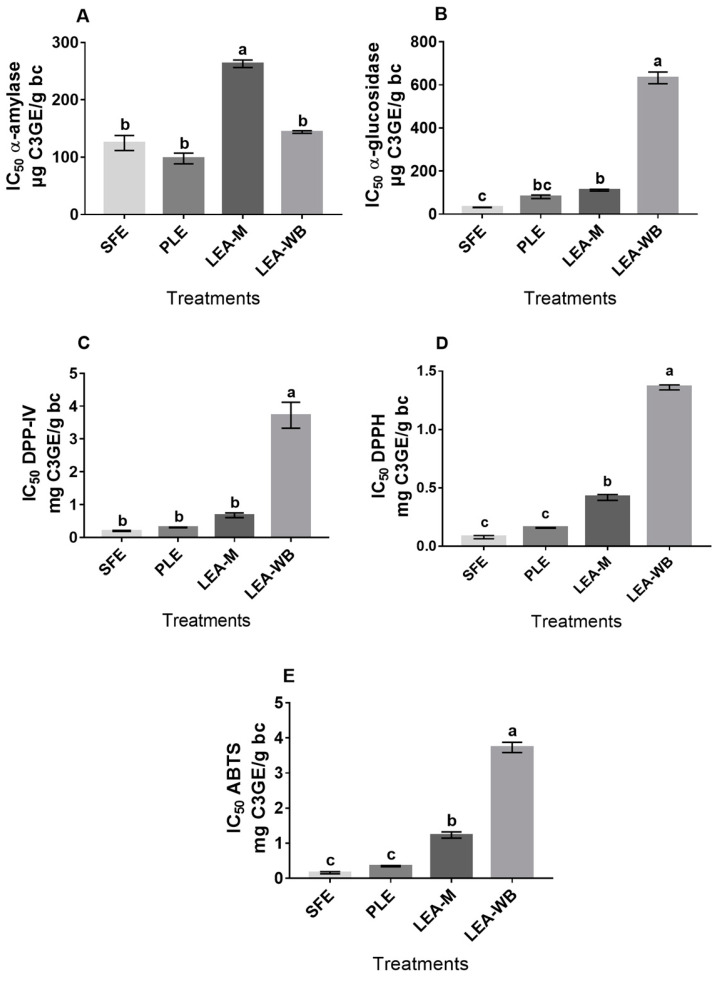
Biological potential of anthocyanin-rich extract from different methods. Half-maximal inhibitory concentration (IC_50_) of α-amylase (**A**), α-glucosidase (**B**), DPP-IV (**C**), DPPH (**D**) and ABTS (**E**). SFE: Supercritical extraction, PLE: Pressurized liquid extraction, LEA-M: Leaching extraction of manually husked bean coat, LEA-WB: Leaching extraction of whole black bean. C3GE: Cyanidin 3-glucoside equivalents, bc: Bean coat. The results are shown as mean ± standard error. Different letters indicate significant differences among samples at *p* < 0.05 (Tukey’s HSD post hoc analysis).

Anthocyanins and other phenolic compounds recovered by SFE and PLE showed the greatest capacity to inhibit the three enzymes, unlike LEA-M and LEA-WB, which were only effective either against α-glucosidase and DPP-IV or against α-amylase, respectively (Figure 2A–C). SFE and PLE extracts had the lowest values of IC_50_, which is the concentration of inhibitor required to inhibit a given biological component by 50%. SFE extracts were two–three times more efficient to inhibit α-amylase, α-glucosidase and DPP-IV than LEA-M.

One of the therapeutic approaches in T2DM treatment is the reduction of the postprandial glycemia through the capacity of phenolic compounds to bind and inhibit the enzymes α-amylase, α-glucosidase and DPP-IV [20]. The inhibition of α-amylase and α-glucosidase is likely to be competitive; the glycosyl group of the anthocyanin binds to the active site, due to the structural similarity of the normal substrate, but it will not be hydrolyzed. Another mechanism is that the hydroxyl groups of the anthocyanin and other phenolic compounds could interact with the polar groups present in the active site of the enzyme, changing its molecular configuration, and therefore, the enzymatic activity [21]. Moreover, phenolic compounds can inhibit DPP-IV activity by binding into the active sites of the enzyme through the interaction of the hydroxyl groups and hydrogen bonds. As a result, the inhibition of DPP-IV avoids the degradation of incretins GLP-1 and GIP, thus improving glucose tolerance in patients with T2DM, by enhancing their insulin-producing effects [22,23].

It has been reported that the inhibition of T2DM related enzymes might be more influenced by individual phenolic compounds, rather than total phenolic compounds [24]. In this regard, phenolic compounds such as myricetin, quercetin, luteolin and rutin are considered as high potent enzyme inhibitors of α-amylase, α-glucosidase and DPP-IV [24,25]. Compared to other phenolic compounds, the aforementioned compounds might have several polar groups with high affinity to the active sites of enzymes, being able to form hydrogen bonds with amino acids and modify their activity [25]. Therefore, the variation in the inhibition capacity of the extracts can be due to the high pressure and temperature used in SFE and PLE, leading to different chemical profiles by recovering phenolic compounds that cannot be extracted by the conventional leaching extraction. In this work, different antidiabetic compounds were identified in SFE and PLE extracts, such as myricetin, quercetin, rutin hydrate and chlorogenic acid [24,25,26]. The high antidiabetic potential found in SFE and PLE extracts might be due to the synergism of these molecules, which provides a unique biological effect. Moreover, there are other non-identified phenolic compounds in a higher proportion than LEA-M, that may have also contributed to the observed biological effect (Table 2) [22].

#### 3.5.2. Antioxidant Potential

DPPH and ABTS assays are based on the direct capture of organic radicals by the antioxidant [27]. As noted in Figure 2D,E, the extracts exhibited greater antioxidant capacity when tested by the ABTS assay, since the steric hindrance of the DPPH nitrogen-centered radical strongly affects the reaction rate of antioxidant compounds [11]. The lowest IC_50_ values were found in the extract obtained by SFE and PLE. The SFE registered an IC_50_ value of 0.078 ± 0.01 mg C3GE/g bean coat against DPPH and 0.161 ± 0.03 mg C3GE/g bean coat against ABTS. For PLE, IC_50_ values of 0.158 ± 0.003 mg C3GE/g bean coat in DPPH method and 0.349 ± 0.01 mg C3GE/g bean coat in ABTS method were found. It has been pointed out that the antioxidant potential is mainly due to their phenolic compounds [28]. The biologic activity of the phenolic compounds is correlated to their structure, including the type, number and position of the substituents. The presence of a catechol group, 3-OH substitutions or the insertion of alkyl groups in the carboxylic acid of flavonoids molecules can lead to enhanced antioxidant potential [29,30]. Those molecules have a high hydrogen donation capacity, due to the increased number of delocalized electrons [31]. Therefore, besides anthocyanins, SFE and PLE could have extracted insoluble-bound phenolics and other phenolic compounds with the aforementioned structural characteristics, which may be responsible for the increased antioxidant potential [14]. The synergism of these molecules could also provide a strong antioxidant effect. Based on the results of antidiabetic and antioxidant potential, our hypothesis is partially supported. The biological potential of SFE and PLE extract was higher than leaching extracts, but it depended on the type of phenolic compounds rather than the total amount of TPC.

### 3.6. Stability of the Anthocyanin-Rich Extracts

The stability of the anthocyanin-rich extract recovered by supercritical CO_2_ was evaluated using the extract obtained from the bean coat through leaching (LEA-M) as control.

#### 3.6.1. Effect of Light Exposure

The regression coefficient indicated that degradation of anthocyanin solutions from both SFE and LEA-M followed first-order reaction kinetics (Figure S2), which is in agreement with other studies [2,5]. The degradation of anthocyanin under different light conditions can be observed in Table 3A.

As expected, the degradation of these compounds was lower under dark condition for both solutions. Values of degradation rate constant (k) decreased in both anthocyanin solution, the lowest k being 0.0221 d^−1^ for the anthocyanin solution by leaching in dark condition. Anthocyanins extracted by leaching were less susceptible to light degradation, when compared to those recovered by SFE, registering lower k and higher half-life values (t_1/2_). Then, for dark condition, the t_1/2_ value of 17.28 days was obtained for anthocyanin solution by LEA-M, while the obtained by SFE was 5.34 days, which is three times lower. Although the degradation of anthocyanins was higher in SFE extract than LEA-M, the degradation (k and t_1/2_) of total phenolic compounds was statistically equal (*p* < 0.05), either in dark or light condition (Table S4A).

As can be seen in Table 3B, the color parameters are correlated with anthocyanin degradation parameters. The lightness (L*) of the anthocyanin solution by LEA-M exhibited a stable behavior, showing differences of L* values less than one. However, solutions made with SFE had their L* values increased, from 90.9 to 95.15 in light condition and 90.9 to 93.48 in dark condition, suggesting color fading. The color parameter a* is associated with red and green on the chromaticity coordinate. Positive values of a* indicate red color, while negative values indicate green color. The a* value in all solutions decreased over time (Table 3), but the solutions by LEA-M were more stable than those by SFE, thus they registered the lowest k and t_1/2_ values of a* (Table S4B). In addition, the effect of light exposure on the change in color (ΔE*) of LEA-M and SFE can be seen in Figure S3. SFE extracts showed a higher ΔE* value than LEA-M, suggesting a faster degradation of the extract. These results are correlated with Table 3 and Table S4 data. The loss of the red color is mainly due to the degradation of anthocyanins. The degradation is likely due to the oxidation and cleavage of covalent bonds of the anthocyanins, which leads to the generation of colorless smaller molecules [2].

The stability of anthocyanins depends on the type of anthocyanin, the phenomenon of co-pigmentation and the other phenolic compounds that may be present. There are anthocyanins that are more susceptible to degradation than other anthocyanins, due to their own structural differences [32]. It has been suggested that the chemical structures of co-pigments are one of the fundamental factors for co-pigmentation interactions [31,32]. The number of delocalized electrons and the structural conformation that a molecule can adopt might determine its efficiency as a co-pigment [8]. In this regard, molecules with large π systems and geometrical flexibility could allow the efficient formation of numerous molecular interactions between the pigment and co-pigment, thus it would not be surprising that hydroxycinnamic acids can act as better co-pigment than hydroxybenzoic acids [8,33]. Therefore, due to the different chemical profile of the extracts, SFE likely could have recovered phenolic compounds that cannot act as efficient co-pigments, due to its own structural limitations, while LEA-M could have extracted compounds that can easily form π-π stacking interactions.

#### 3.6.2. Effect of Storage

Like the experiment of light effect, the regression coefficient indicated that degradation of anthocyanin solutions from both SFE and LEA-M followed first-order reaction kinetics stored at 4 °C, 25 °C and 32 °C (Figure S2).

As shown in Table 4A, the lowest values of k were found in samples stored at 4 °C, but no statistical differences were observed between the anthocyanin solution by LEA-M and SFE for each storage temperature. For t_1/2_, the highest values were found in samples stored at 4°C and in the same case as k, without statistical differences between LEA-M and SFE for each temperature. However, the values of k for TPC in SFE samples were higher than LEA-M, suggesting a faster degradation (Table S5). As expected, the degradation rate constant increases as the storage temperature increases, thus the preservation of anthocyanin results in better storing at lower temperatures. It is well known that temperature is a factor that affects the stability of anthocyanins. Degradation can happen by two mechanisms, the hydrolytic opening of the heterocyclic ring, which leads to the formation of colorless chalcone, and the hydrolysis of the 3-glucoside, to form the unstable aglycone [15].

Q_10_ indicates the change in the reaction rate constant for 10°C. The higher the value, the more temperature-dependent the reaction is [2]. The solution by LEA-M exhibited a more temperature-dependent reaction, while the solution by SFE had lower values of Q_10_ (*p* < 0.05). However, LEA-M solution has a higher Ea than SFE solution. Ea value indicates the energy that the degradation reaction needs. The higher the value is, the more stable the anthocyanin is [2,32].

The effect of storage on color parameters of anthocyanin solutions can be seen in Table 4B. In general, the higher the temperature of storage, the lower the value of a*, thus the color was more stable at a lower temperature. The variation in L* and a* values was higher in SFE solution than in LEA-M solution. Those results are correlated with Figure S4, where the effect of temperature storage on ΔE* values of LEA-M and SFE extracts can be seen. SFE solutions showed a higher ΔE* value than LEA-M in all evaluated temperatures.

Even though there were no statistical differences of k and t_1/2_ between both solutions, the values of Ea, ΔE* and a* suggest the slightly higher stability of anthocyanins extracted by leaching. Since other molecules that could act as co-pigment are not added, the stability of the anthocyanins depends mainly on the interaction with the phenolic compounds, that could have been extracted with the respective methods. Therefore, certain molecules present in the LEA-M extract could co-pigment more efficiently than those recovered with SFE [33]. In the present work, a high proportion of syringic acid and catechin were extracted by LEA-M, which are molecules considered as good co-pigments. These compounds have been reported as better co-pigments than other phenolic compounds, such as gallic acid, chlorogenic acid and epicatechin [33]. Therefore, the presence of a high proportion of efficient co-pigments in the extract by leaching could have preserved the anthocyanin stability in a better way than SFE. Phenolic compounds recovered by SFE might be more susceptible to degradation, limiting the effect of co-pigmentation. However, some of these molecules could be those that exert an important antidiabetic potential. The results of light and storage stability of anthocyanins were not supported for our hypothesis. A higher quantity of phenolic compounds does not improve the stability, since the type of phenolic compound influences the co-pigmentation effect.

## 4. Conclusions

SFE and PLE are alternative extraction methods that have shown an efficient recovery of anthocyanins and other phenolic compounds from black bean. Temperature and co-solvent composition were the most significant factors for extraction. SFE technique decreased solvent consumption and sample processing time. Moreover, the recovered anthocyanin-rich extract showed a different chemical profile, when compared to conventional leaching extraction. Of the eleven non-colored phenolic compounds identified in the SFE anthocyanin-rich extract, syringic acid, chlorogenic acid, catechin, rutin hydrate and myricetin were the main compounds. Anthocyanins and other phenolic compounds from SFE showed an outstanding biological potential, by inhibiting T2DM related enzymes and free radicals, being able to contribute to the prevention and treatment of type 2 diabetes mellitus. In fact, the inhibition of DPP-IV is suggested as the first-line treatment of T2DM, since it is efficient and safe. Nevertheless, further in vivo experiments are needed to confirm and validate the antidiabetic potential of the black bean anthocyanin-rich extract. On the other hand, their stability is still one of the main challenges to overcome. Although SFE extract exhibited the same storage stability as the control, a possible stability improvement can be obtained through the use of co-pigments. The results of this work demonstrated the viability of SFE to obtain high yields of anthocyanins and other phenolic compounds with antioxidant and antidiabetic potential from black bean. Furthermore, since these molecules also have attractive colors, they might be of great interest to the food industry, due to their potential use as a natural colorant.

## Figures and Tables

**Table 1:** Anthocyanins and total phenolic compounds from different extraction methods.

Extraction Method	Anthocyanins	Total Phenolic Compounds
	mg C3GE/g Bean Coat	mg GAE/g Bean Coat
PLE	2.26 ± 0.34 ^a^	7.05 ± 1.32 ^bc^
SFE	2.64 ± 0.53 ^a^	11.09 ± 2.43 ^a^
LEA-M	2.43 ± 0.07 ^a^	8.92 ± 0.3 ^ab^
LEA-WB	1.26 ± 0.05 ^b^	3.15 ± 0.26 ^d^

SFE: Supercritical fluid extraction, PLE: Pressurized liquid extraction, LEA-M: Leaching extraction of manually husked bean coat, LEA-WB: Leaching extraction of whole black bean, C3GE: Cyanidin 3-glucoside equivalents, GAE: Gallic acid equivalents. Different letters indicate significant differences within a column at *p* < 0.05 (Tukey’s HSD post hoc analysis).

**Table 2:** Qualitative analysis of the extracts obtained by different methods.

Peak	Compound	t_R_ (min)	m/z	% Relative Area			
				LEA-M	LEA-WB	SFE	PLE
1	Catechin	0.770	289.1659	15.78	1.12	6.40	1.93
2	Chlorogenic acid	0.779	353.1818	0.29	1.56	13.52	16.28
3	Ferulic acid	0.802	193.0495	1.66	1.84	4.41	6.90
4	Gallic acid *	3.693	169.1064	0.10	0.14	1.97	0.15
5	Synrigic acid	3.806	197.9050	38.65	8.44	11.85	25.04
6	Myricetin-3-O-glucoside *	4.295	479.1749	2.25	0.84	2.87	5.20
7	p-Coumaric acid	4.343	163.1147	0.46	1.85	0.29	0.93
8	Rutin hydrate	4.642	609.2362	1.07	39.40	9.25	9.22
9	Myricetin	5.515	317.1280	18.06	3.35	4.92	4.54
10	Quercetin	6.229	301.1333	7.51	1.56	2.61	2.25
11	Kaempferol	7.311	285.1339	7.08	1.51	3.15	4.05
−	Others	−	−	7.09	38.39	38.75	23.50

LEA-M: Leaching extraction of manually husked bean coat, LEA-WB: Leaching extraction of whole black bean, SFE: Supercritical fluid extraction, PLE: Pressurized liquid extraction. Results are expressed as percentages of the total area, representing the relative abundance of one specific peak related to the sum of the areas of all peaks in the chromatogram for each extraction method. −: non-identified compounds, the percentages were summed and related to the sum of all peaks in the chromatogram. m/z values are related to negative ionization. Compounds with * symbol were identified only with m/z values, while compounds without the symbol were identified with m/z values and pure standards.

**Table:** (**A**)

Parameter	Light Condition	Anthocyanins	
		LEA-M	SFE
Rate (k, d^−1^)	Light	0.0401 ± 0.001 **^aB^**	0.1297 ± 0.006 **^aA^**
	Dark	0.0221 ± 9 × 10^−4^ **^bB^**	0.0824 ± 0.001 **^bA^**
Half-life (t_1/2_, d)	Light	17.28 ± 0.622 **^bA^**	5.34 ± 0.27 **^bB^**
	Dark	31.35 ± 1.305 **^aA^**	8.40 ± 0.12 **^aB^**
Regression coefficient, *R*^2^	Light	0.95	0.99
	Dark	0.94	0.99

**Table:** (**B**)

Extraction Method	Light Condition	L*		a*		b*		Color	
		Initial	Final	Initial	Final	Initial	Final	Initial	Final
LEA-M	Light	91.13	91.94	18.48	13.02	0.37	3.29	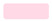	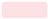
	Dark	91.13	91.36	18.48	15.09	0.37	1.64	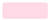	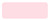
SFE	Light	90.9	95.15	18.98	7.98	−0.67	2.53	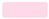	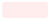
	Dark	90.9	93.48	18.98	11.66	−0.67	1.73	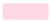	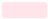

LEA-M: Leaching extraction of manually husked bean coat, SFE: Supercritical fluid extraction, D: Days. Different lowercase letters show significant differences within the same sample under distinct light condition, while different uppercase letters show significant differences among samples under the same light condition (*p* < 0.05, two-sample *t*-test). The results are shown in mean ± standard error.

**Table:** (**A**)

Parameter	Temperature	Anthocyanins	
		LEA-M	SFE
**Rate (k, d^−1^)**	4 °C	0.0038 ± 5 · 10^4 **bA**^	0.0119 ± 9 · 10^−4 **cA**^
	25 °C	0.0226 ± 0.0027 ^**abA**^	0.0306 ± 5 · 10^−4 **bA**^
	32 °C	0.0388 ± 0.0028 ^**aA**^	0.0688 ± 0.0013 ^**aA**^
**Half-life (t_1/2_, d)**	4 °C	188.29 ± 25.65 ^**aA**^	57.83 ± 4.37 ^**aA**^
	25 °C	30.62 ± 3.71 ^**bA**^	22.63 ± 0.384 ^**bA**^
	32 °C	17.81 ± 1.31 ^**bA**^	10.07 ± 0.189 ^**cA**^
**Q_10_**	(4–25 °C)	2.3546 ± 0.046 ^**aA**^	1.8093 ± 0.040 ^**aB**^
	(25–32 °C)	2.1765 ± 0.038 ^**aA**^	1.7134 ± 0.034 ^**aB**^
**Energy of activation,** **Ea (kJ/mol)**		58.7467 ± 1.34 ^**A**^	40.6617 ± 1.53 ^**B**^
**Regression coefficient, *R*^2^**		0.99	0.99

**Table:** (**B**)

Extraction Method	Temperature	L*		a*		b*		Colour	
		Initial	Final	Initial	Final	Initial	Final	Initial	Final
**LEA-M**	4°C	91.13	90.52	18.48	17.57	0.37	−0.07	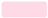	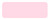
	25°C	91.13	91.77	18.48	12.44	0.37	3.22	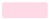	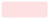
	32°C	91.13	93.12	18.48	8.17	0.37	6.46	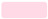	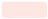
**SFE**	4°C	90.9	92.15	18.98	15.14	−0.67	0.13	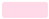	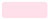
	25°C	90.9	94.98	18.98	7.45	−0.67	2.72	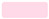	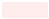
	32°C	90.9	96.58	18.98	4.02	−0.67	3.66	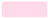	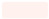

LEA-M: Leaching extraction of manually husked bean coat, SFE: Supercritical fluid extraction, D: days, Q_10_: change in the reaction rate constant for 10 °C. Different lowercase letters show significant differences within the same sample at distinct storage temperature (*p* < 0.05, Tukey HSD post hoc analysis), while different uppercase letters show significant differences among samples under the same storage temperature (*p* < 0.05, two-sample *t*-test). The results are shown in mean ± standard error.

## Supplementary Materials

The following are available online at https://www.mdpi.com/2304-8158/9/5/655/s1, Table S1: D-optimal experiment design matrix for extraction with pressurized liquid (PLE), Table S2. D-optimal experiment design matrix for extraction with supercritical CO_2_ (SFE), Table S3. Comparative table of the recovery of bioactive compounds from common bean by conventional and alternative extraction methods, Table S4. First-order reaction kinetics for (A) total phenolic compounds and (B) color a* parameters degradation of extracts under light and dark conditions after 10 days, Table S5. First-order reaction kinetics and Arrhenius parameters for (A) total phenolic compounds and (B) color a* parameters degradation of extracts at 4 °C, 25 °C and 32 °C after six weeks, Figure S1. Representative UHPLC chromatogram of the anthocyanin extracts and the % relative area (absorbance at 520 nm), Figure S2. Arrhenius plot of anthocyanins at different temperatures for Leaching extraction of manually husked bean coat LEA-M (A) and Supercritical fluid extraction—SFE (B)., Figure S3. Effect of light exposure on the color difference (ΔE*) of anthocyanin-rich extract from LEA-M and SFE, Figure S4. Effect of temperature storage on the color difference (ΔE*) of anthocyanin-rich extract from LEA-M and SFE. (A): 4 °C. (B): 25 °C, (C): 32 °C.
