# Supplementary material for: Black Bean Anthocyanin-Rich Extract from Supercritical and Pressurized Extraction Increased In Vitro Antidiabetic Potential, While Having Similar Storage Stability
Source: Foods. 2020 May 19;9(5):655. doi: 10.3390/foods9050655 (PMC7278739; doi:10.3390/foods9050655)
Supplement: Supplementary file 1 [file foods-09-00655-s001.pdf]

Supplementary Material

Table S1. D-optimal experiment design matrix for extraction with pressurized liquid (PLE)

Run	Pressure	Temperature	Cosolvent	Anthocyanins (mg C3GE/g coat)	Total phenolic compounds (mg GAE/g coat)
1	250	40	EtOH-H_2O 70%	1.14±0.01[j]	3.86±0.35[k]
2	250	40	EtOH-H_2O 70%	0.53±0.006[l]	1.79±0.05[l]
3	100	40	EtOH-H_2O 70%	0.65±0.005[k]	1.89±0.008[l]
4	250	60	EtOH-H_2O 70%	2.61±0.06[a]	11.89±0.14[a]
5	100	40	EtOH-H_2O 70%	0.73±0.008[k]	2.13±0.04[l]
6	100	60	EtOH-H_2O 70%	2.13±0.03[ef]	7.45±0.14[f]
7	100	60	EtOH-H_2O 70%	2.19±0.02[de]	7.39±0.07[fg]
8	200	60	EtOH-H_2O 50%	2.23±0.04[d]	8.77±0.15[d]
9	100	40	EtOH-H_2O 50%	1.28±0.02[i]	4.02±0.05[k]
10	200	40	EtOH-H_2O 50%	1.90±0.008[g]	6.29±0.05[hi]
11	100	60	Acidified H_2O	1.86±0.05[g]	8.85±0.14[d]
12	100	50	Acidified H_2O	1.32±0.03[i]	5.84±0.11[ij]
13	250	60	Acidified H_2O	1.85±0.04[g]	8.38±0.14[de]
14	100	40	Acidified H_2O	0.67±0.005[k]	3.59±0.08[k]
15	200	40	Acidified H_2O	1.08±0.01[j]	5.63±0.18[j]
16	200	50	Acidified H_2O	1.61±0.02[h]	6.82±0.09[gh]
17	100	50	EtOH-H_2O 50%	2.14±0.06[def]	7.91±0.59[ef]
18	250	40	Acidified H_2O	0.75±0.01[k]	4.05±0.09[k]
19	250	50	EtOH-H_2O 50%	2.08±0.02[f]	7.44±0.21[f]
20	200	60	EtOH-H_2O 50%	2.33±0.03[c]	9.72±0.11[c]
21	250	50	Acidified H_2O	1.30±0.007[i]	5.59±0.22[j]
22	250	60	EtOH-H_2O 70%	2.48±0.001[b]	10.43±0.2[b]

EtOH: Ethanol, H_2O: Water, C3GE: Cyanidin 3-glucoside equivalents, GAE: Gallic acid equivalents. Different letters indicate significant differences within a column at $p < 0.05$ (Tukey HSD post hoc analysis).

Table S2. D-optimal experiment design matrix for extraction with supercritical CO_2 (SFE)

Run	Pressure	Temperature	Cosolvent	Anthocyanins (mg C3GE/g coat)	Total phenolic compounds (mg GAE/g coat)
1	300	60	Acidified H_2O	3.15±0.02^{a}	8.81±0.26^{b}
2	200	40	Acidified H_2O	2.48±0.05^{g}	5.56±0.01^{jk}
3	160	40	EtOH-H_2O 50%	2.72±0.03^{de}	7.54±0.16^{e}
4	160	60	EtOH-H_2O 10%	2.29±0.07^{h}	6.00±0.18^{hi}
5	300	60	EtOH-H_2O 50%	2.73±0.04^{de}	8.63±0.16^{bc}
6	200	60	Acidified H_2O	2.63±0.009^{efg}	6.67±0.02^{fg}
7	300	40	EtOH-H_2O 10%	1.98±0.02^{jk}	5.21±0.03^{kl}
8	160	40	EtOH-H_2O 50%	2.78±0.006^{de}	6.93±0.13^{f}
9	160	60	EtOH-H_2O 50%	2.84±0.01^{cd}	9.00±0.04^{b}
10	160	50	Acidified H_2O	2.76±0.08^{de}	6.11±0.07^{h}
11	300	50	Acidified H_2O	2.26±0.05^{hi}	5.49±0.04^{jk}
12	300	40	EtOH-H_2O 50%	2.81±0.06^{cd}	8.33±0.05^{cd}
13	160	40	EtOH-H_2O 10%	2.11±0.02^{ij}	5.48±0.15^{jk}
14	300	60	EtOH-H_2O 10%	3.00±0.02^{ab}	9.67±0.07^{a}
15	300	50	EtOH-H_2O 10%	2.25±0.01^{hi}	6.99±0.12^{f}
16	160	60	EtOH-H_2O 50%	2.95±0.12^{bc}	8.06±0.12^{d}
17	160	60	EtOH-H_2O 10%	2.08±0.04^{j}	6.32±0.05^{gh}
18	160	50	Acidified H_2O	2.30±0.03^{h}	5.67±0.08^{ij}
19	300	40	Acidified H_2O	1.79±0.04^{l}	4.32±0.13^{m}
20	200	50	EtOH-H_2O 10%	2.56±0.05^{fg}	6.77±0.14^{f}
21	160	40	EtOH-H_2O 10%	1.91±0.04^{kl}	5.04±0.07^{l}
22	300	50	EtOH-H_2O 50%	2.65±0.01^{ef}	8.24±0.04^{d}

EtOH: Ethanol, H_2O: Water, C3GE: Cyanidin 3-glucoside equivalents, GAE: Gallic acid equivalents. Different letters indicate significant differences within a column at $p < 0.05$ (Tukey HSD post hoc analysis).

Table S3. Comparative table of the recovery of bioactive compounds from common bean by conventional and alternative extraction methods

	**Time required to process 50 grams of common bean**		
**Method**	**Conventional (Leaching)**	**Alternative (SFE)**	**Alternative (PLE)**
**Husking**	8 h	Does not require sample processing	Does not require sample processing
**Grinding**	30 min		
**Sieving**	30 min		
**Extraction**	15 min	1 h	15 min
**Centrifugation**	15 min	Does not apply	Does not apply
		**Other advantages**	
**Solvent amount**	High (212 mL)	Low (95 mL)	High (212 mL)
**Solvent type**	Ethanol and hydrochloric acid	Ethanol and water	Ethanol and water
**Cotyledon recovery**	Yes	Yes	Yes

SFE: Supercritical fluid extraction, PLE: Pressurized liquid extraction, h: hour(s), min: minute(s).

Table S4. First-order reaction kinetics for (A) total phenolic compounds and (B) color a* parameters degradation of extracts under light and dark conditions after 10 days.

(A)

		Total phenolic compounds	
**Parameter**	**Light condition**	**By LEA-M**	**By SFE**
**Rate (k, d^{-1})**	Light	0.0162 ± 0.002^{aA}	0.0210 ± 0.0002^{aA}
	Dark	0.0090 ± 0.002^{aA}	$0.0167 \pm 1 \cdot 10^{-4bA}$
**Half-life ($t_{1/2}$, d)**	Light	44.09 ± 5.587^{aA}	33.01 ± 0.455^{bA}
	Dark	89.37 ± 24.073^{aA}	41.31 ± 0.353^{aA}
**Regression coefficient, R^2**	Light	0.99	0.95
	Dark	0.98	0.93

(B)

		Color a*	
**Parameter**	**Light condition**	**By LEA-M**	**By SFE**
**Rate (k, d^{-1})**	Light	$0.0349 \pm 5 \cdot 10^{-4\ aB}$	$0.0866 \pm 2 \cdot 10^{-4aA}$
	Dark	$0.0202 \pm 7 \cdot 10^{-5\ bB}$	$0.0487 \pm 4 \cdot 10^{-4bA}$
**Half-life ($t_{1/2}$, d)**	Light	19.82 ± 0.322^{bA}	7.99 ± 0.024^{bB}
	Dark	34.25 ± 0.118^{aA}	14.22 ± 0.141^{aB}
**Regression coefficient, R^2**	Light	0.99	0.99
	Dark	0.99	0.99

LEA-M: Leaching extraction of manually husked bean coat, SFE: Supercritical fluid extraction, D: days. Different lowercase letters show significant differences within the same sample under distinct light condition, while different uppercase letters show significant differences among samples under the same light condition (p-level < 0.05, two-sample t-test). The results are shown in mean ± standard error.

Table S5. First-order reaction kinetics and Arrhenius parameters for (A) total phenolic compounds and (B) color a* parameters degradation of extracts at 4°C, 25°C and 32°C after six weeks.

(A)

		Total phenolic compounds	
**Parameter**	**Temperature**	**By LEA-M**	**By SFE**
**Rate (k, d^{-1})**	4°C	$0.0031\pm1\cdot10^{-4\,bB}$	$0.0061\pm2\cdot10^{-4\,aA}$
	25°C	$0.0051\pm6\cdot10^{-5\,aB}$	$0.0060\pm1\cdot10^{-4\,aA}$
	32°C	$0.0061\pm1\cdot10^{-4\,aA}$	0.0110 ± 0.0015^{aA}
**Half-life ($t_{1/2}$, d)**	4°C	224.71 ± 12.92^{aB}	113.80 ± 3.90^{aA}
	25°C	133.82 ± 1.52^{bB}	114.09 ± 2.11^{aA}
	32°C	113.72 ± 2.87^{bA}	65.07 ± 8.96^{bA}
**Q_{10}**	(4 – 25°C)	1.2817 ± 0.039^{aA}	1.2398 ± 0.078^{aA}
	(25 – 32°C)	1.2527 ± 0.035^{aA}	1.2151 ± 0.070^{aA}
**Energy of activation, Ea (kJ/mol)**		16.63 ± 2.12^{A}	14.47 ± 4.36^{A}
**Regression coefficient, R^2**		0.99	0.99

(B)

		Color a*	
**Parameter**	**Temperature**	**By LEA-M**	**By SFE**
**Rate (k, d^{-1})**	4°C	$0.0011\pm3\cdot10^{-5\,cB}$	$0.0053\pm5\cdot10^{-5\,cA}$
	25°C	$0.0094\pm1\cdot10^{-5\,bB}$	$0.0222\pm7\cdot10^{-5\,bA}$
	32°C	$0.0194\pm5\cdot10^{-5\,aB}$	$0.0369\pm1\cdot10^{-4\,aA}$
**Half-life ($t_{1/2}$, d)**	4°C	580.68 ± 16.28^{aA}	128.99 ± 1.20^{aB}
	25°C	73.55 ± 1.52^{bA}	31.13 ± 0.094^{bB}
	32°C	35.69 ± 0.094^{bA}	18.77 ± 0.095^{cB}
**Q_{10}**	(4 – 25°C)	2.74 ± 0.031^{aA}	2.09 ± 0.010^{aB}
	(25 – 32°C)	2.50 ± 0.026^{aA}	1.88 ± 0.009^{bB}
**Energy of activation, Ea (kJ/mol)**		69.33 ± 0.795^{A}	47.87 ± 0.371^{B}
**Regression coefficient, R^2**		0.99	0.99

LEA-M: Leaching extraction of manually husked bean coat, SFE: Supercritical fluid extraction, D: days, Q_{10}: change in the reaction rate constant for 10°C. Different lowercase letters show significant differences within the same sample at distinct storage temperature (p-level < 0.05, Tukey HSD post hoc analysis), while different uppercase letters show significant differences among samples under the same storage temperature (p-level < 0.05, two-sample t-test). The results are shown in mean ± standard error.

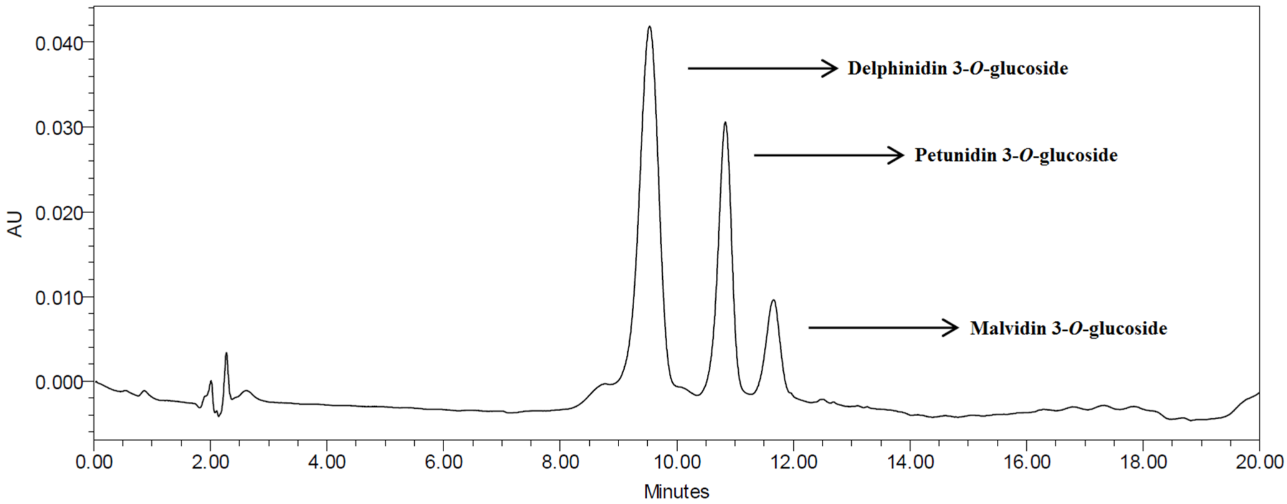

Anthocyanin	Retention time (min)	% relative area
Delphinidin 3-*O*-glucoside	9.488	58.37±0.24
Petunidin 3-*O*-glucoside	10.806	30.92±0.40
Malvidin 3-*O*-glucoside	11.635	10.69±0.22

Figure S1. Representative UHPLC chromatogram of the anthocyanin extracts and the % relative area (absorbance at 520 nm). The results are shown in mean ± standard deviation.

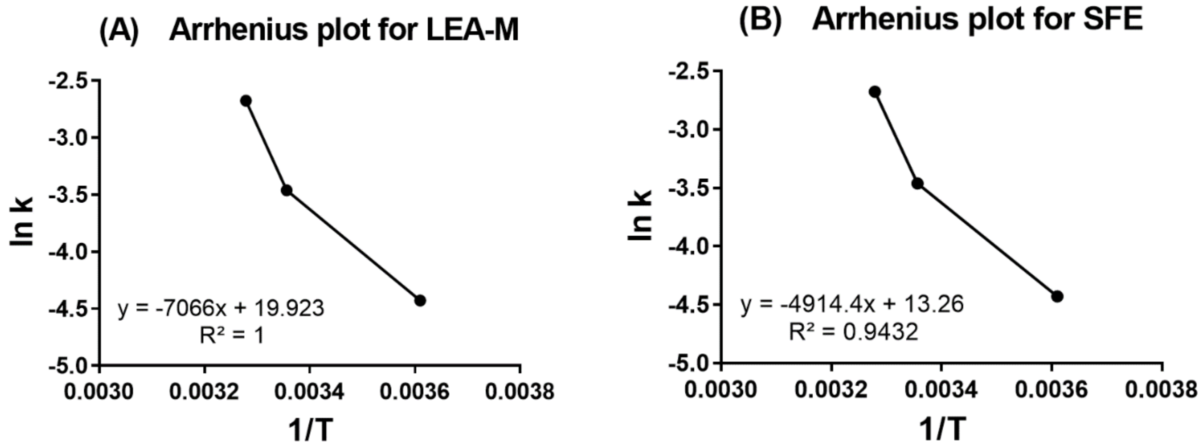

Figure S2. Arrhenius plot of anthocyanins at different temperatures for Leaching extraction of manually husked bean coat LEA-M (A) and Supercritical fluid extraction – SFE (B). k: Rate, T: Temperature, R^2: Regression coefficient.

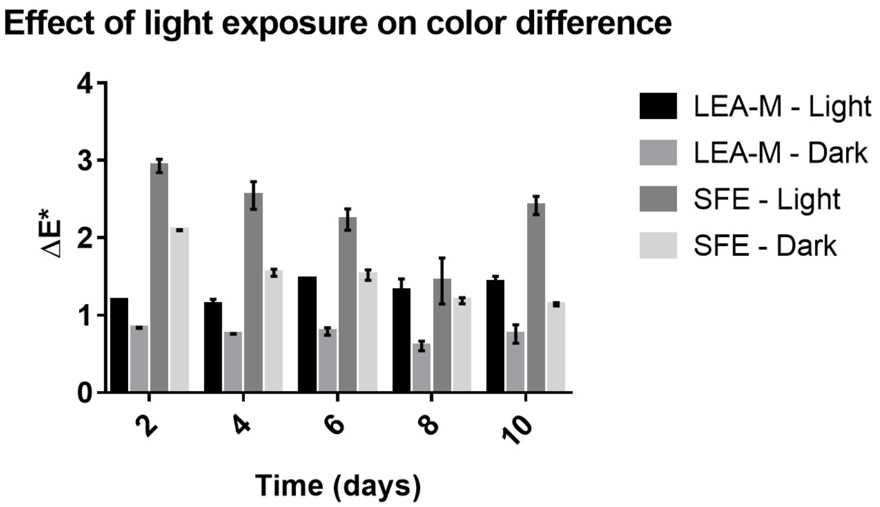

Figure S3. Effect of light exposure on the color difference (ΔE*) of anthocyanin-rich extract from LEA-M and SFE. LEA-M: Leaching extraction of manually husked bean coat, SFE: Supercritical fluid extraction. The results are shown in mean ± standard error.

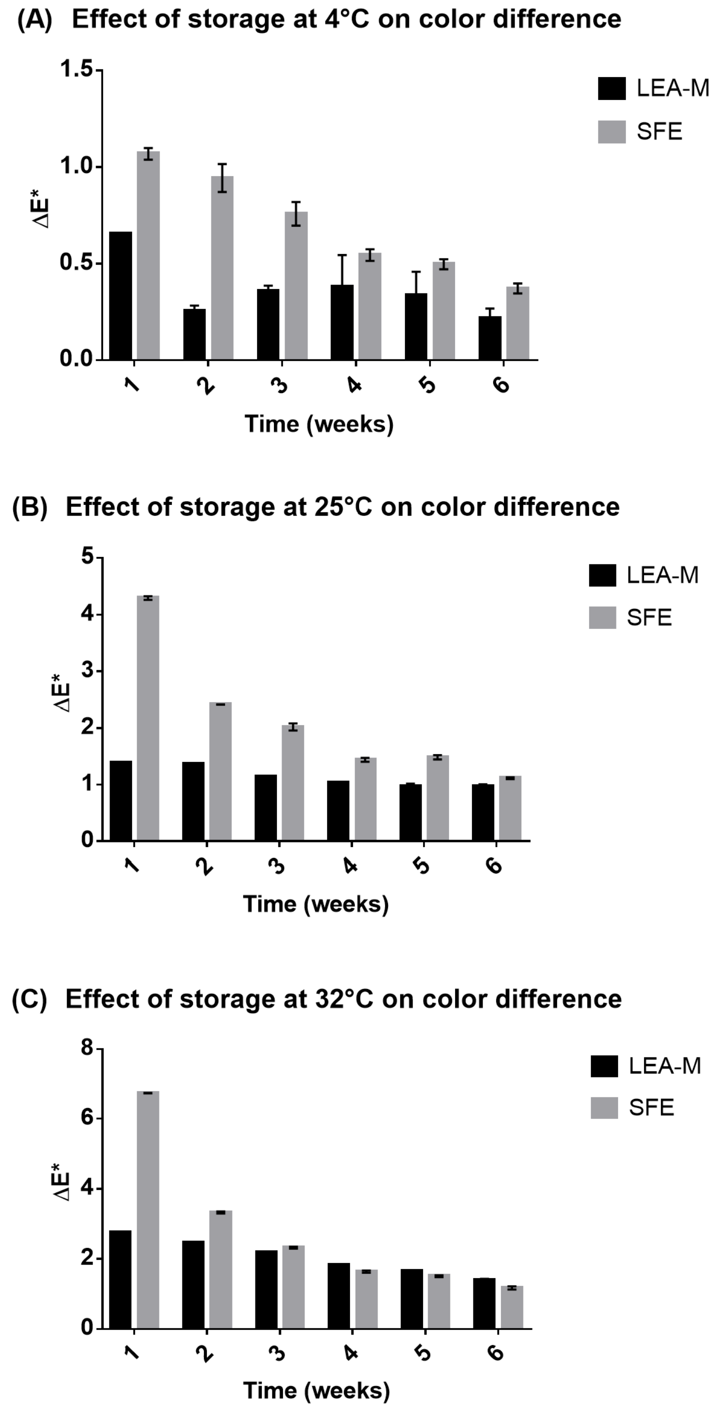

Figure 4. Effect of temperature storage on the color difference (ΔE*) of anthocyanin-rich extract from LEA-M and SFE. (A): 4°C. (B): 25°C, (C): 32°C. LEA-M: Leaching extraction of manually husked bean coat, SFE: Supercritical fluid extraction. The results are shown in mean ± standard error.
